# Variations in host genes encoding adhesion molecules and susceptibility to falciparum malaria in India

**DOI:** 10.1186/1475-2875-7-250

**Published:** 2008-12-04

**Authors:** Swapnil Sinha, Tabish Qidwai, Kanika Kanchan, Prerna Anand, Ganga N Jha, Sudhanshu S Pati, Sanjib Mohanty, Saroj K Mishra, Prajesh K Tyagi, Surya K Sharma, Indian Genome Variation Consortium, Vimala Venkatesh, Saman Habib

**Affiliations:** 1 Division of Molecular and Structural Biology, Central Drug Research Institute, Post box 173, Chattar Manzil, Mahatma Gandhi Marg, Lucknow-226001, India; 2 King George Medical University (KGMU), Lucknow, India; 3 Ispat General Hospital, Rourkela, India; 4 National Institute of Malaria Research Field Station, Rourkela, India; 5 Institute of Genomics and Integrative Biology, Delhi, India

## Abstract

**Background:**

Host adhesion molecules play a significant role in the pathogenesis of *Plasmodium falciparum *malaria and changes in their structure or levels in individuals can influence the outcome of infection. The aim of this study was to investigate the association of SNPs of three adhesion molecule genes, *ICAM1*, *PECAM1 *and *CD36*, with severity of falciparum malaria in a malaria-endemic and a non-endemic region of India.

**Methods:**

The frequency distribution of seven selected SNPs of *ICAM1*, *PECAM1 *and *CD36 *was determined in 552 individuals drawn from 24 populations across India. SNP-disease association was analysed in a case-control study format. Genotyping of the population panel was performed by Sequenom mass spectroscopy and patient/control samples were genotyped by SNaPshot method. Haplotypes and linkage disequilibrium (LD) plots were generated using PHASE and Haploview, respectively. Odds-ratio (OR) for risk assessment was estimated using EpiInfo™ version 3.4.

**Results:**

Association of the ICAM1 rs5498 (exon 6) G allele and the CD36 exon 1a A allele with increased risk of severe malaria was observed (severe versus control, OR = 1.91 and 2.66, P = 0.02 and 0.0012, respectively). The CD36 rs1334512 (-53) T allele as well as the TT genotype associated with protection from severe disease (severe versus control, TT versus GG, OR = 0.37, P = 0.004). Interestingly, a SNP of the *PECAM1 *gene (rs668, exon 3, C/G) with low minor allele frequency in populations of the endemic region compared to the non-endemic region exhibited differential association with disease in these regions; the G allele was a risk factor for malaria in the endemic region, but exhibited significant association with protection from disease in the non-endemic region.

**Conclusion:**

The data highlights the significance of variations in the *ICAM1*, *PECAM1 *and *CD36 *genes in the manifestation of falciparum malaria in India. The *PECAM1 *exon 3 SNP exhibits altered association with disease in the endemic and non-endemic region.

## Background

Severe clinical outcomes from *Plasmodium falciparum *infection have been associated with cytokine imbalance and high levels of pro-inflammatory cytokines such as TNF, high parasitaemia due to the failure of the host immune system to control parasite replication, acidosis and respiratory distress, as well as sequestration of infected RBCs (iRBCs) in the microvasculature. Several host molecules have been implicated in mediating cerebral and non-cerebral cytoadherence of *P. falciparum*-infected RBCs to the host endothelium [1], a process that helps the parasite evade immune clearance in the spleen. Host adhesion molecules including CD36, intercellular adhesion molecule 1 (ICAM-1, CD54), platelet/endothelial cell adhesion molecule 1 (PECAM-1, CD31), vascular cell adhesion molecule (VCAM-1), thrombospondin, E-selectin, P-selectin, chondroitin sulphate A and FcRs may serve as receptors for ligands such as PfEMP1, expressed on the surface of iRBCs to mediate cytoadherence [2]. The precise role of some of these molecules in mediating cytoadherence *in vivo *remains unresolved due to the observation of minimal to no adhesion to these receptors when using patient isolates or under flow conditions [3-5]. Molecules such as ICAM-1 and VCAM-1 are inducible (e.g. by TNF) and their expression levels are elevated during acute malaria infection [6]. As important components of the host immune system host cell adhesion molecules may also play a role in parasite-induced immune regulation. Alterations in levels or structure of these molecules would thus affect an individual's response to *P. falciparum *infection and consequent disease manifestation.

Susceptibility/resistance of human populations to severe falciparum malaria has been associated with variations in more than 30 genes. Polymorphisms in host adhesion molecules have been correlated with the outcome of *P. falciparum *infection in studies from Africa and south-east Asia. Single nucleotide polymorphisms (SNPs) in genes encoding CD36, ICAM-1 and PECAM-1 have been previously correlated with disease protection/susceptibility from different populations but these reports have often been contradictory. A mutation in codon 29 (K56M, rs5491) of the *ICAM1 *gene was identified in the Kilifi region of Kenya and homozygotes for the mutation were found to be more frequent in patients suffering from cerebral malaria than in controls [7]. However, this SNP was correlated with protection from severe malaria in Gabon [8] and did not exhibit any association with disease severity in Gambia [9]. Additionally, an ICAM-1 SNP in exon 6 (rs5498, K469E) has been associated with increased risk of severe malaria in Nigeria [10]. A recent study [11] on trios from African populations has reported the absence of any association of these two ICAM-1 SNPs with severe malaria phenotypes.

Two SNPs in the CD36 gene, T1264G in exon 10 and G1439C in exon 12, that encode truncated CD36 proteins and are the molecular basis of CD36 deficiency were found in high frequency in Kenyan and Gambian patients suffering from severe malaria [12] while another study on patients from Kenya reported association of the T1264G heterozygote with protection from severe malaria in children [13]. Variation screening of the CD36 gene in Thai malaria patients revealed that two SNPs in the promoter region at positions -53 and -14 were associated with protection from cerebral malaria together with a repeat polymorphism (TG)_12 _in intron 3 that was strongly associated with reduced risk of cerebral malaria [14].

Mutant homozygotes for two non-synonymous SNPs (L125V and S563N) of the *PECAM1 *gene have been reported to be one of the risk factors for cerebral malaria in Thailand [15]. However, no association of the L125V SNP with disease was reported in a study on malaria patients from Kenya and Papua New Guinea [16].

As evident from the above description, all available data for disease-SNP correlation for adhesion molecules is for populations from Africa and Thailand. *P. falciparum *malaria is a serious problem in India and several regions of the country are endemic for the disease [17,18]. The distribution of selected SNPs of genes encoding CD36, ICAM-1 and PECAM-1 was thus examined in Indian populations and SNP-disease association analysis of seven SNPs from the three genes was carried out in a case-control study with patients and ethnically-matched controls drawn from a *P. falciparum*-endemic and a non-endemic region of India.

## Methods

### Populations, study subjects and sample collection

Allele frequency distribution of selected SNPs from genes encoding *ICAM1*, *PECAM1 *and *CD36 *was carried out in the existing Indian Genome Variation Consortium (IGVC) panel II. This panel consisted of 552 samples drawn from 24 ethnically and linguistically diverse populations belonging to various tribal, caste and religious groups from different geographical regions of India. Panel II was derived from the initial IGVC sample set of 1871 individuals from 55 populations (Panel I) [19]. The population descriptors included linguistic affiliation (Indo-European, IE; Dravidian, DR; Tibeto-Burman, TB; Austro-Asiatic AA) followed by geographical zone (North, N; North-East, NE; South, S; East, E; West, W; Central, C) and ethnicity (caste, LP; tribe, IP; religious group, SP) (description of each population is available at [20]). A population of known African descent was included as an outgroup population (OG-W-IP).

For the case control study, patient samples were collected from a *P. falciparum *endemic (Antagarh, Chhattisgarh and Sundargarh, Orissa) and a non-endemic (Lucknow and surrounding areas of Uttar Pradesh) region of India. The uninfected controls were ethnically-matched with the patient group and belonged to the Bhumij, Munda, Oraon and Gond tribal populations in the endemic region and the Aggarwal, Brahmin, Kayastha, Pasi, Thakur, Yadav, Shia and Sunni caste and religious groups in the non-endemic region. Informed consent was obtained from each volunteer/guardian prior to collection. 2–5 ml of venous blood was drawn from patients of above five years of age diagnosed with *P. falciparum *malaria. Diagnosis was carried out by rapid diagnostic test kits (Optimal/Paracheck) followed by confirmation by examination of thick and thin blood smears. In rare cases of discrepancy between the results of the two tests, *P. falciparum *infection was confirmed by a diagnostic polymerase chain reaction (PCR) [21]. WHO guidelines [22] were followed to categorize severe and non-severe malaria as described in Sinha *et al *[23]. Samples from a total of 182 *P. falciparum *malaria patients (101 from endemic and 81 from non-endemic region) were collected. Control samples were collected from ethnically-matched and unrelated individuals from the endemic (102 samples) and non-endemic (90 samples) regions. This study has been approved by ethical committees of all participating institutes.

### Selection of SNPs and genotyping

SNPs were selected according to their reported functional relevance in disease, including falciparum malaria, in other world populations as well as their frequency in the IGVC 'discovery panel'. The 'discovery panel' of 43 samples was used for an initial screen for discovery of novel SNPs and validation of reported polymorphisms in Indian populations [19]. The following SNPs from *ICAM1*, *PECAM1*, *CD36 *and *VCAM1 *were genotyped in the IGVC validation panel samples: rs5491, K/M (*ICAM1*); rs668, L/V (*PECAM1*); rs12953, S/N (*PECAM1*); rs1131012, R/G (*PECAM1*); int10 novel SNP, G/A (*PECAM1*); int15, rs2070783 C/T (*PECAM1*); exon10 rs3211938, Y/STOP (*CD36*); exon12, A/P (*CD36*); rs2151916, -14 T/C (*CD36*); rs1334512, -53 G/T (*CD36*); rs3783611, exon5 C/T (*VCAM1*); rs3783613, exon6 G/C (*VCAM1*); rs2392221, int3 C/T (*VCAM1*); rs3176860, int2 A/G (*VCAM1*). For the case-control study, non-synonymous SNPs rs5491 and rs5498 of *ICAM1 *and rs668, rs12953 and rs3211938 of *PECAM1 *were genotyped. The novel *PECAM1 *intron 10 SNP (nt33289, ref seq NT_010783) was discovered in the initial panel of 'discovery' samples [24]. Three SNPs of *CD36*, -14 T/C (upstream promoter region), -53 G/T (downstream promoter region) and exon 1a T/A [14], were used for the association study.

Genomic DNA was extracted from peripheral blood leukocytes using salting-out procedure [25]. Genotyping of the IGVC panel samples was performed by Sequenom mass spectroscopy. Genotyping of patient/control samples was done by SNaPShot method (Applied Biosystems) on an ABI 3130xl automated DNA sequencer.

### Statistical analysis

The chi-square test was performed to evaluate whether the allele frequencies of populations are in Hardy-Weinberg equilibrium. Haplotypes and linkage disequilibrium (LD) plots were generated using PHASE and Haploview, respectively. Odds-ratio (OR) for risk assessment was estimated using EpiInfo™ version 3.4 software programme which calculates P-value by Fisher exact or Mantel-Haenszel test.

## Results and discussion

### Distribution of selected SNPs of ICAM1 (CD54), PECAM1 (CD31) and CD36 genes in Indian populations

In order to understand population-specific allele frequency distribution of SNPs from genes encoding adhesion molecules CD36, ICAM-1 and PECAM-1, genotype data from Panel I and Panel II of IGVC populations was analysed. The *ICAM1 *rs5491 SNP frequency was estimated for 55 IGVC populations of Panel I [19]. SNPs were selected according to their reported functional relevance in disease, including falciparum malaria, in other world populations as well as their frequency in the IGVC 'discovery panel' that was used for an initial screen for discovery of novel SNPs and validation of reported polymorphisms [19].

**Figure 1:**
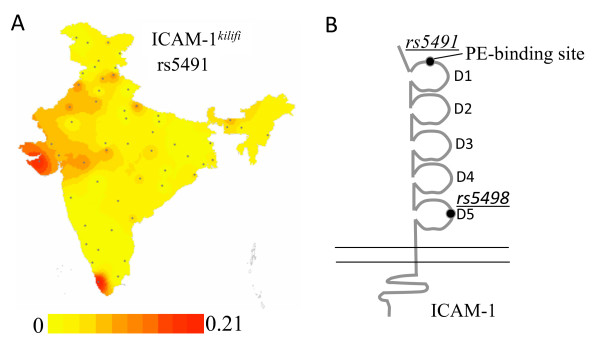
**(A) Gradient map showing frequency distribution of the minor allele of ICAM-1 rs5491 (A/T) in 55 Indian populations.** Dots on the map depict the location (site of collection) of the 55 populations. (B) Schematic representation of the ICAM-1 molecule showing location of the amino acid changes due to rs5491 and rs5498.

The *ICAM1 *rs5491 K/M (*ICAM1*^*Kilifi*^) SNP, previously associated with disease response in Africa [7,8], has an average frequency of 0.042 in the Indian population which is much lower than its frequency in Yoruba (YRI, MAF = 0.25) and similar to the Han Chinese and Japanese (HCB and JPT, MAF = 0.08) populations of HAPMAP. Ten Indian populations lacked the polymorphism while maximal SNP frequency was observed in the outgroup population of known African descent (OG, MAF = 0.18) and a tribal population of southern India (DR-S-IP1, MAF = 0.23) (Figure 1a). Low frequency of this SNP has also been reported for some other tribal populations of India [26]. Very low frequencies of the SNP were observed in populations of both the *P. falciparum *malaria endemic (MAF = 0.04) and non-endemic region (MAF = 0.02) and the SNP was thus excluded from the disease association analysis. The *ICAM1 *exon 6 A/G (rs5498, K/E) SNP, previously correlated with susceptibility to severe malaria in Nigeria [10], exhibited high frequency in the control group (MAF = 0.39) and was included in the case-control study.

**Figure 2:**
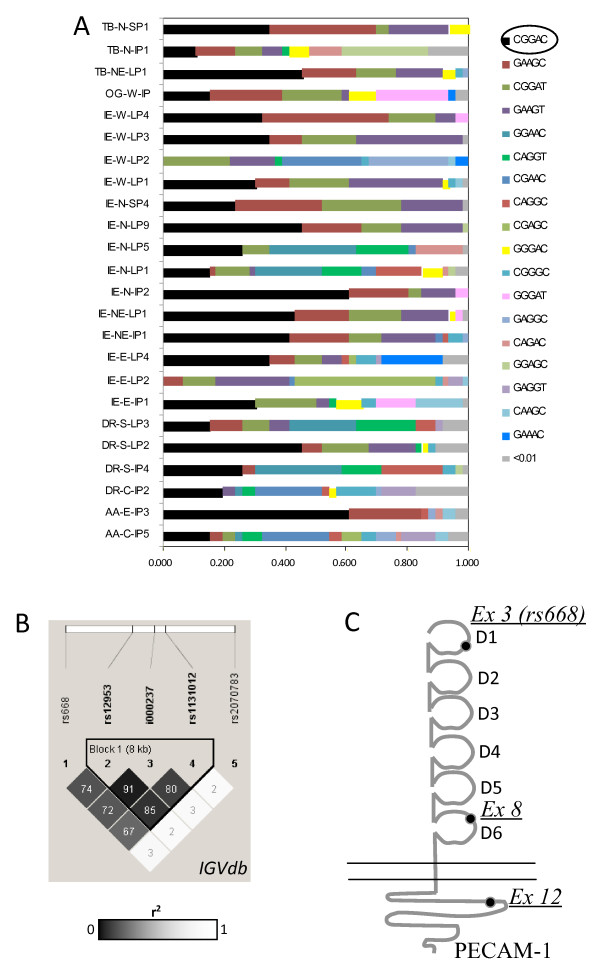
**(A) Distribution of *PECAM1 *haplotypes for the SNPs rs668, rs12953, Int10 (novel SNP, i000237), rs1131012 and rs2070783 across 24 Indian populations.** The wild-type haplotype is circled. (B) R^2 ^LD plot of the five *PECAM1 *SNPs. The value in each cell is the % D' between SNP pairs; grayscale depicts r^2 ^values. (C) Schematic representation of the PECAM-1 molecule showing location of the amino acid changes due to SNPs in exon 3 (rs668), exon 8, and exon 12.

Five SNPs of the *PECAM1 *gene including the exon 3 rs668 L/V, exon 8 rs12953 S/N, and exon 12 rs1131012 R/G previously associated with malaria as well as clinical complications [27,28] and two intronic SNPs (intron10 novel SNP G/A, intron15 rs2070783 C/T) were analysed. All SNPs had high frequency in India with average MAFs ranging from 0.33 to 0.45. The exon 8, intron 10, and exon 12 SNPs were in strong LD and formed a haplotype block of ~8 kb in the *PECAM1 *gene (Figure 2b). PECAM1 showed high haplotype diversity (mean haplotype diversity = 0.778) which was >70% in most populations. A total of 29 haplotypes were generated by PHASE among which 18 haplotypes had a frequency > 0.01 (Figure 2a). The wild haplotype CGGAC (Ex3 C/G, Ex8 G/A, Int10 G/A, Ex12 A/G, Int15 C/G) had the highest frequency (29.5%). However, this was not the predominant haplotype in some populations. Other major haplotypes were GAAGC (13.5%), CGGAT (11.6%) and GAAGT (11.5%). These either carry the wild type sequence at Ex8, Int10 and Ex12 or have the mutated bases at all the three positions again revealing strong linkage between these SNPs.

The exon 10 rs3211938, Y/STOP and exon12 A/P SNPs of the CD36 genes have been previously correlated with protection from severity and susceptibility to severe *P. falciparum *malaria, respectively in African populations (Kenya and Gambia) [12,13]. However, both these SNPs were found to be monomorphic in the 24 Indian populations of Panel II. The -14 T/C and -53 G/T SNPs from the upstream and downstream promoter of the gene had average MAFs of 0.41 and 0.43, respectively in Indian populations. The two promoter SNPs, together with the 5'UTR (exon 1a) T/A SNP [14] were analysed for disease association.

Of the four VCAM-1 SNPs genotyped in Panel II, the two non-synonymous SNPs in exon 5 and exon 6 (rs3783611 and rs3783613) were found to be monomorphic in Indian populations. Very low frequency of these SNPs has been reported from other Asian populations as well. The two intronic SNPs, rs2392221 and rs3176860, had a MAF of 0.13 and 0.46, respectively. None of the VCAM-1 SNPs were analysed for disease-association.

### A non-synonymous polymorphism in domain 5 of ICAM-1 is a risk factor for severe malaria

The *ICAM1 *rs5498 (A/G) SNP lies in the exon 6 region encoding Ig-like domain 5 of the extracellular portion of the ICAM-1 molecule (Figure 1b). The mutant allele G was associated with risk of severe malaria in combined analysis of patients and controls from the endemic and non-endemic region [severe versus control, odds ratio (OR) = 1.91, P = 0.02 95% CI = 1.05–3.49; severe versus non-severe OR = 1.99, P = 0.016, 95% CI = 1.09–3.65; non-severe versus control, OR = 0.96, P = 0.88, 95% CI = 0.52–1.76] (Table 1). Although the GG genotype was associated with severity when severe patients were compared with controls (GG & AA: OR = 3.45, P = 0.0004) no significant difference in distribution of the GG genotype was observed between severe and non-severe malaria patients (GG & AA: OR = 0.93, P = 0.87). These results indicate association of the *ICAM1 *rs5498 G allele with risk of severe malaria in India. Since ICAM-1 is the major host molecule implicated in cytoadherence of iRBCs in cerebral malaria [29], severe malaria cases were stratified into cerebral and non-cerebral malaria. No significant difference in GG genotype distribution was observed in the two severe patient groups indicating that the SNP was not specifically associated with manifestation of cerebral malaria.

**Table 1:** Genotype frequency distribution of SNPs in patient and control groups

Gene name	SNP	Control (n = 192)	Non-severe (n = 89)	Severe (n = 93)
***ICAM1***	**Ex6 (rs5498)**			
	AA	70 (36.5%)	17 (19.1%)	21 (22.5%)
	AG	93 (48.4%)	46 (51.7%)	42 (45.2%)
	GG	29 (15.1%)	26 (29.2%)	30 (32.3%)

***PECAM1***	**Ex3 (rs668)**			
	CC	108 (56.3%)	39 (43.8%)	43 (46.2%)
	CG	53 (27.6%)	36 (40.4%)	40 (43%)
	GG	31 (16.1)	14 (15.7%)	10 (10.8%)
	
	**Ex8 (rs12953)**			
	GG	80 (41.7%)	30 (33.7%)	40 (43%)
	AG	76 (39.5)	40 (44.9%)	32 (34.4%)
	AA	36 (18.8%)	19 (21.3%)	21 (22.6%)
	
	**Ex12 (rs1131012)**			
	AA	69 (35.9%)	28 (31.4%)	41 (44.1%)
	AG	101 (52.6%)	43 (48.3%)	34 (36.6%)
	GG	22 (11.5%)	18 (20.2%)	18 (19.3%)

***CD36***	**-14 (rs2151916)**			
	TT	83 (43.2%)	31 (34.8%)	38 (40.9%)
	TC	76 (39.6%)	42 (47.2%)	38 (40.9%)
	CC	33 (17.2%)	16 (18%)	17 (18.2%)
	
	**-53 (rs1334512)**			
	GG	61 (31.8%)	34 (38.2%)	47 (50.5%)
	GT	87 (45.3%)	36 (40.4%)	32 (34.4%)
	TT	44 (22.9%)	19 (21.3%)	14 (15.1%)
	
	**Ex1a**			
	TT	115 (59.9%)	42 (47.2%)	38 (40.9%)
	TA	59 (30.7%)	32 (36%)	30 (32.2%)
	AA	18 (9.4%)	15 (16.8%)	25 (26.9%)

The *ICAM1 *exon 6 (K/E, rs5498) SNP has been correlated with many inflammatory and neurodegenerative diseases [30-32] and has been identified as a risk factor for severe malaria in Nigerian children [10]. Results from this study also indicate association of the minor G allele with increased risk of severe malaria. Domain 5 of the ICAM-1 molecule, that harbours the K/E mutation, has been reported to affect the dimerization of ICAM-1 [33] as well as interaction between B cells and dendritic cells [34]. It has been suggested that dimerization of ICAM-1 enhances its binding to its natural ligand LFA-1 [35]. The residue important for binding of ICAM-1 to the parasite-encoded ligand PfEMP1 lies in the dimer interface of domain 1 but whether binding involves monomeric or dimeric ICAM-1 is still unknown [33]. The exon 6 mutation changes the basic amino acid residue lysine to glutamic acid. This mutation may play a role in the destabilization of domain 5 and affect ICAM-1 dimerization thus influencing the binding of ICAM-1 to its ligands. Further, the exon 6 SNP is located 3 bp upstream to the splice donor site that produces the alternately spliced soluble isoform of ICAM-1 (sICAM-1) [36]. Iwao *et al*. [37] have shown that sICAM-1 expression is lowered in cells carrying the GG genotype. Since sICAM-1 does not contain the trans-membrane domain and is secretory in nature it would reduce cell-cell interactions. Further studies would be required to investigate the possible involvement of the exon 6 SNP in ICAM-1 dimerization and its effect on cell-cell interactions that could in turn influence host immune response against malaria.

### The PECAM-1 exon 3 SNP exhibits differential association with disease in the endemic and non-endemic region

Of the *PECAM1 *SNPs analysed for disease association, no significant association was observed with the exon 8 G/A and exon 12 A/G SNPs (Table 1). The exon 3 C/G SNP lies in the region encoding the Ig-like domain 1 of the PECAM-1 molecule (Figure 2c). Although the exon 3 SNP did not exhibit significant association with disease in the combined analysis for the endemic and non endemic region samples, significant association was observed when the data was stratified on the basis of disease endemicity. In the endemic region, the mutant allele G was associated with susceptibility to disease with high frequency of the allele in both severe and non-severe patients as compared to controls (severe versus control, OR = 4.21, P < 0.0001, 95% CI = 2.11–8.44; non-severe versus control, OR = 2.79, P = 0.0016, 95% CI = 1.39–5.69; severe versus non-severe, OR = 1.51, P = 0.15, 95% CI = 0.83–2.75). On the other hand, the G allele was protective in the non-endemic region (severe versus control: OR = 0.4, P = 0.002, 95% CI = 0.21–0.75; non-severe versus control, OR = 0.51, P = 0.02, 95% CI = 0.27–0.96; severe versus non-severe, OR = 0.78, P = 0.43, 95% CI = 0.4–1.51).

PECAM-1 mediated adhesion is complex as the molecule is capable of binding both to itself (homophilic binding) and to other ligands (heterophilic binding) [38]. The *PECAM1 *exon 3 mutation lies in the first Ig-like domain of the PECAM-1 molecule (Fig. 2c) which has been shown to mediate hemophilic adhesion and regulate leukocyte transmigration [39,40]. Treutiger *et al *[41] have identified PECAM-1 as an endothelial receptor for *P. falciparum *infected erythrocytes and this heterophilic adhesion is mediated by domains 1–4 of the PECAM-1 molecule. An antibody directed against domains 1–2 has been shown to inhibit PECAM-1-dependent adhesion of iRBCs *in vitro *[41] possibly due to disruption of interaction with PfEMP1 in iRBCs. The precise functional consequence of the exon 3 L/V mutation is still unknown but if it affects homophilic binding of PECAM-1 then it may have an impact on leukocyte transmigration at the inflammatory site during disease condition. The reported role of PECAM-1 domain 1 in adhesion of iRBCs also suggests a possible role of the exon 3 SNP in affecting iRBC sequestration. It is interesting that the G allele which is a risk factor in the endemic region also had very low frequency in endemic region controls (MAF = 0.18) compared to controls of the non-endemic region (MAF = 0.47) where it is protective. Additionally, the SNP had an average MAF of 0.4 in the 24 Indian populations of IGVC.

### Two CD36 SNPs associate with protection from severe malaria

Since the two *CD36 *SNPs (exon 10 and exon 12) previously associated with *P. falciparum *malaria in Africa were found to be monomorphic in India, the *CD36 *-14 T/C, -53 G/T and exon 1a T/A SNPs were analysed for disease correlation. Although the -14 SNP has been correlated with protection from severe malaria in Thailand [14], no significant correlation was observed between the -14 SNP and disease in this study. As in the Thailand study, the -53 mutant T allele was associated with protection from severe malaria in India with significant difference in its distribution in the severe patient and control groups (severe versus control, T & G, OR = 0.53, P = 0.03, 95% CI = 0.29–0.98). A trend towards higher frequency of the T allele in the non-severe patient group compared to severe patients was observed (severe versus non-severe, T & G, OR = 0.65, P = 0.14, 95% CI = 0.3–1.02) while no significant difference was seen between the non-severe cases and controls (OR = 0.82, P = 0.47). The -53 TT genotype was also significantly associated with protection from severity (severe versus control, TT & GG, OR = 0.37, P = 0.004, 95% CI = 0.16–0.77; severe vs. non-severe, OR = 0.53, P = 0.13, 95% CI = 0.22–1.3; non-severe versus control, OR = 0.83, P = 0.56). The mutant allele A of the *CD36 *exon 1a SNP was a risk factor, particularly for severe malaria (severe versus control, OR = 2.66, P = 0.0012, 95% CI = 1.4–5.07; non-severe versus control, OR = 1.84, P = 0.048, 95% CI = 0.96–3.53; severe versus non-severe, OR = 1.45, P = 0.119, 95% CI = 0.79–2.64). The AA genotype was also associated with increased susceptibility to disease with stronger correlation observed with severe malaria (severe versus control: OR = 4.02, P = 0.00056, 95% CI = 1.89–8.6; severe versus non-severe, OR = 1.84, P = 0.122, 95% CI = 0.79–4.3; non-severe versus control, OR = 2.18, P = 0.04, 95% CI = 0.95–5).

During falciparum malaria, CD36 is not only involved in sequestration of iRBCs but also plays a role in host pathology by inhibiting of dendritic cell maturation [42] as well as host immunity by mediating phagocytic clearance of parasites by macrophages [43]. CD36 is also involved in platelet-mediated clumping of iRBCs that is strongly associated with cerebral malaria [44]. The expression of CD36 is tightly regulated to ensure its unique functions in various cell types. CD36 expression is regulated by two promoters (upstream and downstream) which bind to different transcriptional activators [45]. The -53 G/T mutation that correlates with protection from severe malaria is positioned at the downstream promoter of the *CD36 *gene. This SNP is located in the promoter region that is necessary for basal transcription of the gene [46]. Also, the -53 position lies within the consensus binding site for the transcriptional activator AP2 [47]. However, the actual effect of the -53 SNP on CD36 expression is not known. The exon 1a T/A SNP was reported by Omi *et al *[14] in Thailand but its correlation with disease severity has been shown for first time in this study. The functional significance of the SNP is unclear.

## Conclusion

As part of a study to investigate the role of human genetic factors in susceptibility/resistance to *P. falciparum *malaria in India, the frequency of SNPs in selected adhesion molecules was analysed followed by a case-control study to determine possible association with disease. The results indicate the significance of specific genetic variants of *ICAM1*, *PECAM1 *and *CD36 *in influencing the outcome of falciparum malaria in Indian populations. Additionally, altered association of the *PECAM1 *exon 3 SNP was observed with disease in the endemic and non-endemic region and the allele was differentially distributed in populations of the two regions.

## Abbreviations

ICAM1: intercellular cell adhesion molecule 1; PECAM1: platelet endothelial cell adhesion molecule 1; VCAM1: vascular cell adhesion molecule 1; MAF: minor allele frequency; LD: linkage disequilibrium

## Authors' contributions

SH and VV conceived and designed the study and contributed to execution of research. SH and SS wrote the manuscript. SS, TQ and KK carried out genotyping and data analysis. SS, TQ, GNJ and PA carried out sample collection and processing. SKS helped in implementation of field study. SSP, SM, SKM and PT are clinicians who contributed to diagnosis and categorization of patients. The Indian Genome Variation Consortium (IGVC) comprised scientists from six institutes of the Council of Scientific and Industrial Research with Dr. Samir K. Brahmachari as coordinator. Collection of population samples across India and Sequenom genotyping was coordinated under IGVC. SH is a member of the consortium. All authors have read and approved the final manuscript.
